# Baicalin suppresses NLRP3 inflammasome and nuclear factor-kappa B (NF-κB) signaling during *Haemophilus parasuis* infection

**DOI:** 10.1186/s13567-016-0359-4

**Published:** 2016-08-08

**Authors:** Shulin Fu, Lei Xu, Sali Li, Yinsheng Qiu, Yu Liu, Zhongyuan Wu, Chun Ye, Yongqing Hou, Chien-An Andy Hu

**Affiliations:** 1 Hubei Key Laboratory of Animal Nutrition and Feed Science, Wuhan Polytechnic University, Wuhan, 430023 People’s Republic of China; 2 Hubei Collaborative Innovation Center for Animal Nutrition and Feed Safety, Wuhan, 430023 People’s Republic of China; 3 Biochemistry and Molecular Biology, University of New Mexico School of Medicine, Albuquerque, NM 87131 USA

## Abstract

*Haemophilus parasuis* (*H. parasuis*) is the causative agent of Glässer’s disease, a severe membrane inflammation disorder. Previously we showed that Baicalin (BA) possesses anti-inflammatory effects via the NLRP3 inflammatory pathway in an LPS-challenged piglet model. However, whether BA has anti-inflammatory effects upon *H. parasuis* infection is still unclear. This study investigated the anti-inflammatory effects and mechanisms of BA on *H. parasuis*-induced inflammatory responses via the NF-κB and NLRP3 inflammasome pathway in piglet mononuclear phagocytes (PMNP). Our data demonstrate that PMNP, when infected with *H. parasuis,* induced ROS (reactive oxygen species) production, promoted apoptosis, and initiated transcription expression of IL-6, IL-8, IL-10, PGE_2_, COX-2 and TNF-α via the NF-κB signaling pathway, and IL-1β and IL-18 via the NLRP3 inflammasome signaling pathway. Moreover, when BA was administrated, we observed a reduction in ROS production, suppression of apoptosis, and inhibition of the activation of NF-κB and NLRP3 inflammasome signaling pathway in PMNP treated with *H. parasuis*. To our best knowledge, this is the first example that uses piglet primary immune cells for an *H. parasuis* infection study. Our data strongly suggest that BA can reverse the inflammatory effect initiated by *H. parasuis* and possesses significant immunosuppression activity, which represents a promising therapeutic agent in the treatment of *H. parasuis* infection.

## Introduction

*Haemophilus parasuis* (*H. parasuis*), is the causative agent of Glässer’s disease, whose typical clinical characteristics include polyarthritis, fibrinous polyserositis and meningitis [1]. In recent years, *H. parasuis* has become one of the most important pathogens of livestock worldwide and has caused gross economic losses owing to the expensive antibiotic treatment and the high mortality in piglets [2]. Fifteen serovars of *H. parasuis* have been identified so far [3]. *H. parasuis* is a normal inhabitant of the upper respiratory tract. However, under certain circumstances, such as sudden changes of the external environment, decreasing immunity, etc., virulent strains may replicate and cause pneumonia and other infections [4, 5]. Proliferation of *H. parasuis* in the host cell could lead to a strong inflammatory immune response [6], but so far the mechanisms induced by *H. parasuis* induced inflammation are not exactly clear. Therefore, the inflammatory immune response mediated by *H. parasuis* has become the focus of current research.

A previous study has shown that *H. parasuis* or its cell wall lipooligosaccharides can initiate innate immune response and induce the production of inflammatory cytokines IL-6 and IL-8 in porcine brain microvascular endothelial cells (PBMEC) and tracheal cells [7, 8]. In addition, *H. parasuis* can also activate the inflammatory transcription factor, nuclear factor-kappa B (NF-κB), in a time and dose-dependent manner and cause the release of key inflammatory mediators including IL-8, and CCL4, in PK-15 cells [9, 10]. Interestingly, NF-κB regulates the transcription and expression of IL-8 and CCL4 [11]. Furthermore, *H. parasuis* induces the porcine bone marrow dendritic cells to produce IL-6 and IL-10 [12]. Taken together, it is suggested that cytokines involved in the host innate immune and inflammatory responses and their expressions are regulated by NF-κB.

Recently, it has been proposed that the inflammasome plays an important role in the regulation of bacterial and sterile inflammation [13]. The best-characterized inflammasome, NLRP3, was identified and shown to induce the production of IL-1β and IL-18 during the inflammatory processes [14]. NLRP3 interacts with the adaptor molecule and apoptosis-associated speck-like protein, which harbors a caspase recruitment domain that can be used to recruit and activate caspase-1 [15–17]. Previously, it has been shown that NLRP3 exists primarily in immune and inflammatory cells which are activated by inflammatory stimuli such as LPS [18, 19]. The immune and inflammatory cells include peripheral blood mononuclear cells [18, 20], macrophages [21], conventional splenic neutrophils and dendritic cells [22]. Although innate adaptive immune response can efficiently protect the animal from certain diseases, inappropriate activation of the NLRP3 inflammasome can lead to progression of various diseases [16, 23]. For example, the activation of NLRP3 inflammasome induces renal inflammation that results in chronic kidney disease [24]. Therefore, we are interested in whether NLRP3 inflammasome is involved in *H. parasuis*-induced Glässer’s disease.

Baicalin (BA), is a plant-derived flavonoid from *Scutellaria baicalensis Georgi* (Huang Qin), and its chemical structure has been verified [25]. BA has been shown to possess antioxidant, anti-bacterial, anti-inflammatory and free radical scavenging activities [26, 27]. BA also exhibits anti-influenza virus A (H1N1) activity in vitro and in vivo as a potent inducer of IFN-γ in major IFN-producing cells [28], inhibits dengue virus replication following virus internalization by *vero* cells [29] and suppresses the development of *Candida albicans* biofilms by inducing cell death via apoptosis [30]. BA has been shown to induce apoptosis in human HepG2 and SMMC-7721 cells and significantly inhibit the growth of xenografts in nude mice [31]. The anti-inflammatory properties of BA have been posed by preventing NF-κB signaling pathway in HBE16 airway epithelial cells resulting from the inhibition of IL-6, IL-8, and TNF-α expression [32], inhibiting the Th17 response and reducing silica-induced inflammation and fibrosis [33], protecting keratinocytes from UVB-induced inflammatory damage through TLR pathway modulation [34] and decreasing the iNOS protein expression, inflammatory factors and oxidative stresses in a rat model of acute myocardial infarction [27]. However, the anti-inflammatory mechanism of BA in treating inflammatory diseases of pigs, such as Glässer’s disease, has not been characterized.

Our previous studies demonstrated that the activation of NF-κB and NLRP3-caspase-1 signal pathway were induced by LPS in PMNP and BA was related to the suppression of NLRP3 inflammasome pathway under LPS stimulation [18, 19]. However, the activation of NF-κB and NLRP3-caspase-1 pathway mediated by *H. parasuis* in PMNP and the effects of BA on *H. parasuis* induced activation of the NF-κB and NLRP3 inflammasome have not been investigated. Therefore, to evaluate the effects and the mechanism responsible for the anti-inflammatory activities of BA, we conducted the experiments using PMNP evoked by *H. parasuis*.

## Materials and methods

### Bacterial strain, growth conditions and drugs

The *H. parasuis* SH0165 strain, which is a highly virulent strain of serovar 5, was isolated from the lung of a commercial pig with arthritis, fibrinous polyserositis, hemorrhagic pneumonia and meningitis. The SH0165 was grown in Tryptic soy broth (TSB; Difco Laboratories, USA) supplemented with 10 μg/mL of NAD (Sigma, USA) and 10% newborn calf serum (Gibco, USA) under 37 °C.

Baicalin was obtained from the National Institute for Food and Drug Control (Beijing, B110715-201318). BA was dissolved and diluted using RPMI-1640 medium (Gibco, New York, USA).

### Isolation and culture of peripheral blood monocytes

This study was carried out in strict accordance with the recommendations in the China Regulations for the Administration of Affairs Concerning Experimental Animals 1988 and the Hubei Regulations for the Administration of Affairs Concerning Experimental Animals 2005. The protocol was approved by China Hubei Province Science and Technology Department (permit number SYXK (ER) 2010-0029). All experimental animals were euthanized at the end of the experiments.

Three 35-day-old naturally farrowed, early-weaned (NFEW) piglets (Duroc × Landrace × large white) weighing 7-10 kg which were negative for detection of antibody against *H. parasuis* by INGEZIM Haemophilus 11.*H. parasuis*. K1 (Ingezim, Spain) obtained from Wuhan Cofco Meat Product Co., Ltd (Wuhan, China), were used for in vitro experiments.

Isolation and culture of peripheral blood monocytes (PMNP) was successfully established in our lab previously with some minor modifications [19]. Briefly, heparinized blood from the precaval vein was layered carefully on an equal volume of PBS (pH 7.4) in a conical centrifuge tube, and then carefully layered an equal volume of mixed blood on the surface of lymphocyte separation medium. The suspension was centrifuged at 400 × *g* for 20 min at 25 °C. The cells of the lymphocyte layer were collected and washed three times with PBS, centrifuged at 400 × *g* for 20 min under 4 °C. Then the cells were resuspended in RPMI-1640 medium (Gibco, New York, USA) and seeded in a 6-well cell culture plate (costar, New York, USA). Three milliliters of suspension was added in each well, then these were pre-incubated in a constant temperature incubator at 37 °C with 5% CO_2_ for 3 h in RPMI-1640 containing 10% fetal bovine serum (FBS, Gibco, Australia). The cells were washed three times with PBS and then washed with pre-warmed RPMI-1640 medium (Gibco) to discard the nonadherent cells. Attached cells (monocytes) were detached using a cell scraper and suspended in RPMI-1640 medium (Gibco). Mononuclear cells were counted and their viability was determined by Trypan blue exclusion.

### Dosing schedule effect on blood monocyte viability in vitro

Blood monocyte viability was determined using the cell counting kit-8 (CCK-8) assay [35]. Briefly, monocytes were seeded into 96-well plates at 1 × 10^5^ cells/well and then treated with the baicalin at final concentrations (0, 12.5, 25, 50, 100, 200, 400 μg/mL) for 3, 6, 12, 20 h. Then 10 μL CCK-8 (Dojindo Molecular Technologies, Japan) was added to each well and incubated for 2 h at 37 °C. The absorbance was determined at 450 nm. The monocyte viability was calculated using the following formula: cell viability (%) = (experimental well − blank well/control well − blank well) × 100%. The dates were expressed as mean ± SD of triplicate samples from at least three independent experiments.

### Blood monocyte infection model of *H. parasuis*

In order to explore the multiplicity of infection (MOI) of *H. parasuis* with the monocytes, 5 × 10^5^ cells were seeded into the culture plates. *H. parasuis* was inoculated into 100 mL TSB supplemented with NAD (10 μg/mL) (Sigma) and 10% newborn calf serum (Gibco) and propagated overnight at 37 °C. The bacterial suspension was then diluted 100-fold into fresh TSB and was cultured at 37 °C for 12 h to obtain the log-phase bacteria. The log-phase bacteria were diluted with TSB for further study. Then *H. parasuis* (10^5^, 10^6^, 10^7^ CFU/mL) was added to each well and incubated under 5% CO_2_ at 37 °C for 3, 6, 12, and 20 h, respectively. Inflammatory cytokines from the supernatant were measured to determine the MOI and optimal interaction time.

### Detection of reactive oxygen species (ROS) and cell apoptosis

Intracellular ROS was detected using DCFH-DA staining [36]. Cells (1 × 10^6^) were seeded into 24-well plates and treated with various concentrations of baicalin (25, 50, 100 μg/mL) for 2 h. Then 1 × 10^6^ CFU/mL *H. parasuis* were added into the wells and incubated for 3 and 6 h respectively. Hence the incubations were washed three times with PBS and stained with 10 μM DCFH-DA and 5 μM DHE (ROS, Nanjing Jiancheng Bioengineering Institute, Nanjing, China; apoptosis, NeoBioscience, Shenzhen, China) for 30 min, respectively. The fluorescence intensities were observed by Fluorescence microscopy (Olympus, Japan).

### Determination of cytokine concentrations

In brief, 5 × 10^5^ cells were seeded into 24-well plates and pre-treated with a final concentration of baicalin of 25, 50, 100 μg/mL for 2 h. Then 1 × 10^6^ CFU/mL *H. parasuis* were added into the wells and incubated for 6 h respectively. Hence the supernatants from the cells were collected and centrifuged at 400 × *g* for 15 min under 4 °C. Cytokine concentration in the cell culture supernatants was measured by ELISA assays (porcine IL-1β, IL-18, TNF-α, IL-6, IL-8, IL-10, PGE2, and COX-2, R&D, USA) according to the manufacturers.

### Total RNA extraction and RT-PCR

In order to determine the expression levels of inflammatory cytokines (IL-1β, IL-18, TNF-α, IL-6, IL-8 and IL-10) and NLRP3 Inflammasome (NLRP3, ASC, and Caspase-1) in the infection of monocytes, 1 × 10^7^ of cells were seeded into 24-well plates and pre-treated with 25, 50, 100 μg/mL baicalin for 2 h. Then 1 × 10^7^ CFU/mL *H. parasuis* were added into the wells and co-incubated for 3 and 6 h respectively. Hence the cells were collected and total cellular RNA was extracted from monocytes using the TRISOL reagent (Invitrogen, USA). Then the RNA was reverse-transcribed to cDNA using reverse transcriptase (TaKaRa, Dalian, China) and the amplification of cDNA was determined with the SYBE Green PCR Kit (ABI, USA). Individual transcripts in each sample were repeated three times and β-actin was used as the internal control. Nucleotide sequences of the primers used for Q-PCR are listed in Table 1.

**Table 1:** **Primers for qRT-PCR**

Gene	Nucleotide sequence (5′–3′)	Tm (°C)	Length (bp)
β-actin	Forward TGCGGGACATCAAGGAGAAG	57.4	216
	Reverse AGTTGAAGGTGGTCTCGTGG	57.4	
NLRP3	Forward GGAGGAGGAGGAAGAGGAGATA	59.5	147
	Reverse AGGACTGAGAAGATGCCACTAC	57.7	
ASC	Forward ACAACAAACCAGCACTGCAC	55.4	126
	Reverse CTGCCTGGTACTGCTCTTCC	59.5	
Caspase-1	Forward GAAGGAGAAGAGGAGGCTGTT	57.6	268
	Reverse AGATTGTGAACCTGTGGAGAGT	55.8	
IL-1β	Forward TCTGCATGAGCTTTGTGCAAG	55.6	225
	Reverse ACAGGGCAGACTCGAATTCAAC	57.7	
IL-18	Forward AGTAACCATCTCTGTGCAGTGT	55.8	155
	Reverse TCTTATCATCATGTCCAGGAAC	53.9	
TNF-α	Forward CGCTCTTCTGCCTACTGCACTTC	61.3	164
	Reverse CTGTCCCTCGGCTTTGACATT	57.6	
IL-6	Forward CCAGGAACCCAGCTATGAAC	57.4	142
	Reverse CTGCACAGCCTCGACATT	54.9	
IL-8	Forward CAGAGCCAGGAAGAGACT	54.9	461
	Reverse GACCAGCACAGGAATGAG	54.9	
IL-10	Forward GCATCCACTTCCAGGCCA	57.2	176
	Reverse CTTCCTCATCTTCATCGTCA	53.4	
COX-2	Forward CTGTCCCATCCCTCGGTTTA	54.4	105
	Reverse TCTCTGAGCACTGTCCGTAAT	54.4	

### NF-κB p65 nuclear translocation studies by ELISA

The levels of monocyte p65 were measured to investigate the effects of baicalin pretreatment on *H. parasuis*-activated monocytes NF-κB signaling. The cells of 1 × 10^7^ seeded on 6-well plates were pretreated with baicalin (25, 50, 100 μg/mL) for 2 h. Then 1.0 × 10^7^ CFU/mL *H. parasuis* was added into the wells. After stimulation of 6 h, the cells were collected and cytoplasmic protein and nucleoprotein were extracted with a cytosolic—nuclear protein extraction kit (Beyotime Biotechnology, Shanghai, China). Protein concentrations were determined with the bicinchoninic acid (BCA) protein assay reagents (Beyotime Biotechnology) according to the manufacturer’s instructions. The expression of NF-κB p65 in cytoplasmic protein and nucleoprotein was measured by NF-κB ELISA Kit (Blue Gene Biotechnology, Shanghai, China) and the results of nuclear translocation of NF-κB p65 are presented by nuclear protein expression of NF-κB p65/cytoplasmic protein expression of NF-κB p65.

### Western blot analysis

The 3 × 10^7^ monocyte cells were pretreated with baicalin (25, 50, 100 μg/mL) for 2 h and then 3.0 × 10^7^ CFU/mL *H. parasuis* was added into the wells. After co-culture for 5 h, the cells were collected and then the cell protein was extracted using a total protein extraction kit (Beyotime Biotechnology) according to the manufacturer’s instructions. Hence the protein concentration was measured with the BCA protein assay kit (Sigma, USA). The proteins were isolated by 12% SDS-PAGE and then transferred onto the PVDF membrane. The PVDF membrane was blocked with 5% skim milk at 25 °C for 3 h. After being washed three times with TBST, the PVDF membrane was incubated with cleaved caspases-1 antibody or β-actin antibody (Cell Signaling Technology, USA) for 12 h at 4 °C. Afterwards being washed three times with TBST, the membrane was incubated with HRP-linked goat anti-rabbit antibody at 25 °C for 3 h and visualized using ECL solution (Thermo Pierce ECL, USA). The levels of cleaved caspase-1 and β-actin were detected with the FluorChem FC2 AIC system (Alpha Innotech, USA).

### Statistical analysis

The experimental data were expressed as mean ± SD. The difference between two groups was analyzed using the two-tailed Student’s *t* test. A *p* value of ≤0.05 was considered to indicate a statistically significant result. *p < 0.05 and **p < 0.01.

## Results

### Effect of baicalin on blood monocyte viability in vitro

**Figure 1:**
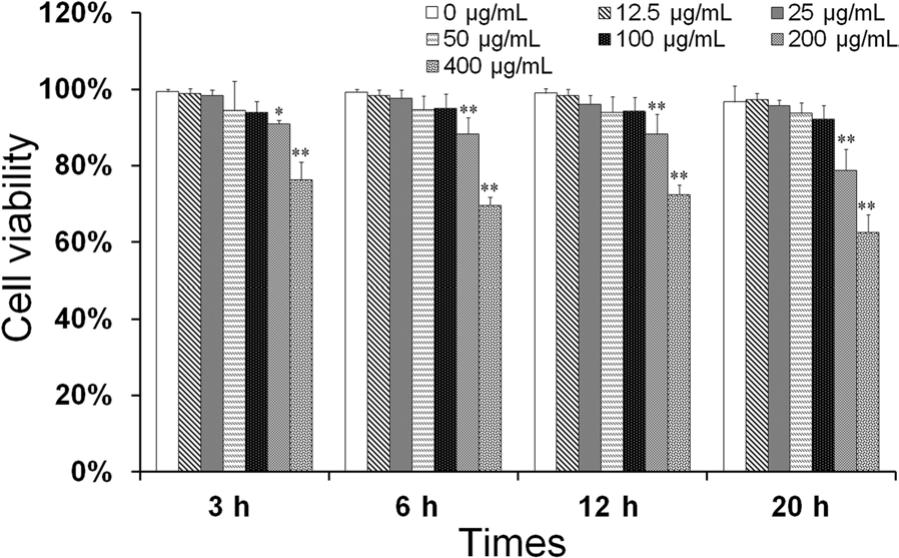
**Effect of baicalin on blood monocyte viability in vitro.** Monocytes were seeded into 96-well plates at 1 × 10^5^ cells/well and then treated with the baicalin at final concentrations (0, 12.5, 25, 50, 100, 200, 400 μg/mL) for 3, 6, 12, 20 h, 10 μL. CCK-8 was added to each well and incubated for 2 h at 37 °C. The absorbance was determined at 450 nm. The date are expressed as mean ± SD of triplicate samples from at least three independent experiments. **indicates significance at *p* < 0.01, *indicates significance at *p* < 0.05.

To optimize the concentration of baicalin, the monocyte viability assay was utilized to evaluate drug-induced toxicity. As the concentration of baicalin decreased from 100 to 12.5 μg/mL, monocyte viability increased from 92.1 to 98.2% and those concentrations did not induce significant cytotoxicity (*p* > 0.05) (Figure 1). Furthermore, the expected correlation ship between the cell viability, drug concentration and incubation time was observed (Figure 1). Thus, the concentration of 100 μg/mL of baicalin was considered as a safe dose and could be used for the next studies.

### Establishing an infection model for porcine peripheral blood monocytes of *H. parasuis*

**Figure 2:**
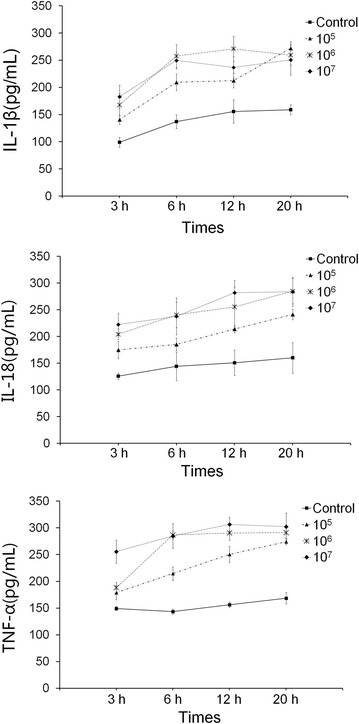
**Establishing a model of infection of porcine** ***peripheral blood monocytes*** **by** ***H. parasuis.*** 5 × 10^5^ cells were seeded into the culture plates, then *H. parasuis* (10^5^, 10^6^, 10^7^ CFU/mL) was added to each well and incubated under 5% CO_2_ at 37 °C for 3, 6, 12, and 20 h, respectively. Inflammatory cytokines from the supernatant were measured to determine the MOI and optimal interaction time.

An infection model was established in this study to determine the optimal MOI between *H. parasuis* and porcine peripheral blood monocytes. When the MOI was 1:5, the production of IL-1β, TNF-α and IL-18 did not increase significantly compared with the control cells, but displayed an upward trend (*p* > 0.05) (Figure 2). When the MOI was 2:1 and 20:1 and the cells were stimulated for 6 h, the concentration of IL-1β, TNF-α and IL-18 in the cell culture supernatant increased compared with the control cells (*p* < 0.01) (Figure 2). Therefore we chose a concentration of 1.0 × 10^6^ CFU/mL of *H. parasuis* and co-culture time at 6 h as the infection model of inflammatory response.

### Effect of baicalin on the reduction of proinflammatory cytokines induced by *H. parasuis* in peripheral blood monocytes

**Figure 3:**
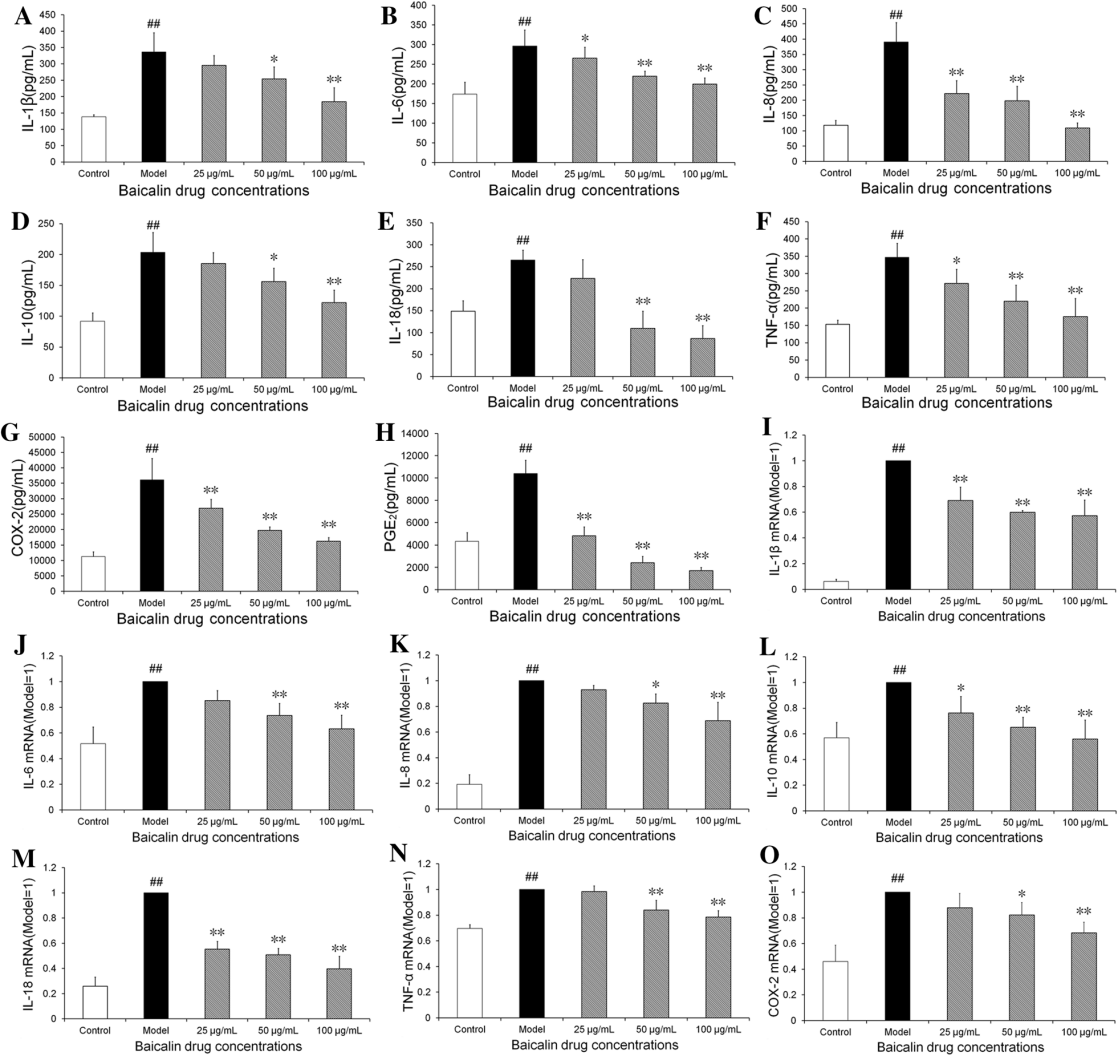
**Effect of baicalin on the reduction of proinflammatory cytokines induced by** ***H. parasuis*** **in peripheral blood monocytes.** **A**–**H** 5 × 10^5^ cells were seeded into 24-well plates and pre-treated with a final concentration of baicalin of 25, 50, 100 μg/mL for 2 h. Then 1 × 10^6^ CFU/mL *H. parasuis* were added into the wells and incubated for 6 h respectively. Hence the supernatants from the cells were collected and centrifuged at 400 × *g* for 15 min under 4 °C. Cytokine concentration in the cell culture supernatants was measured by ELISA assays. **I**–**O** For RNA determination, 1 × 10^7^ of cells were seeded into 24-well plates and pre-treated with baicalin at 25, 50, 100 μg/mL for 2 h. Then 1 × 10^7^ CFU/mL *H. parasuis* were added into the wells and co-incubated for 3, 6 h respectively. Hence the cells were collected, then the total cellular RNA was extracted and was reverse-transcribed to cDNA. Individual transcripts in each sample were repeated three times and β-actin was used as the internal control. The white bar represents the control group. The black bar represents the model group. The twilled bars represent the baicalin-pretreated groups. ^#^ *p* < 0.01 vs. control. **indicates significance at *p* < 0.01, *indicates significance at *p* < 0.05.

To examine *H. parasuis*-induced cytokine secretion, peripheral blood monocytes were infected with *H. parasuis* for 6 h and the levels of IL-6, IL-8, IL-10, PGE_2_, COX-2, IL-1β, IL-18, and TNF-α in the culture media were determined by ELISA. The results show that the levels of IL-6, IL-8, IL-10, PGE_2_, COX-2, IL-1β, IL-18, and TNF-α in the cell culture were markedly increased in the cells stimulated with *H. parasuis* compared with the control (*p* < 0.05) (Figures 3A–H). On the contrary, pretreatment of baicalin (50, 100 μg/mL) significantly decreased the levels of IL-6, IL-8, PGE_2_ and COX-2 in the cell culture in a concentration-dependent manner (*p* < 0.01) (Figures 3B, C, G, H). At the same time the levels of mRNA expression of IL-6, IL-8, IL-10, COX-2, IL-1β, IL-18, and TNF-α were also tested by qRT-PCR. The results demonstrate that the level of mRNA expression of IL-6, IL-8, IL-10, COX-2, IL-1β, IL-18, and TNF-α were significantly upregulated in peripheral blood monocytes following infection with *H. parasuis* for 3 or 6 h (*p* < 0.01) (Figures 3I–O). Meanwhile the levels of mRNA expression of IL-6, IL-8, IL-10, COX-2, IL-1β, IL-18, and TNF-α were significantly inhibited in the cells pretreated by baicalin (50, 100 μg/mL) compared with the model (*p* < 0.05) (Figures 3I–O).

### Effect of baicalin on the production of reactive oxygen species (ROS) and cell apoptosis induced by *H. parasuis* in peripheral blood monocytes

**Figure 4:**
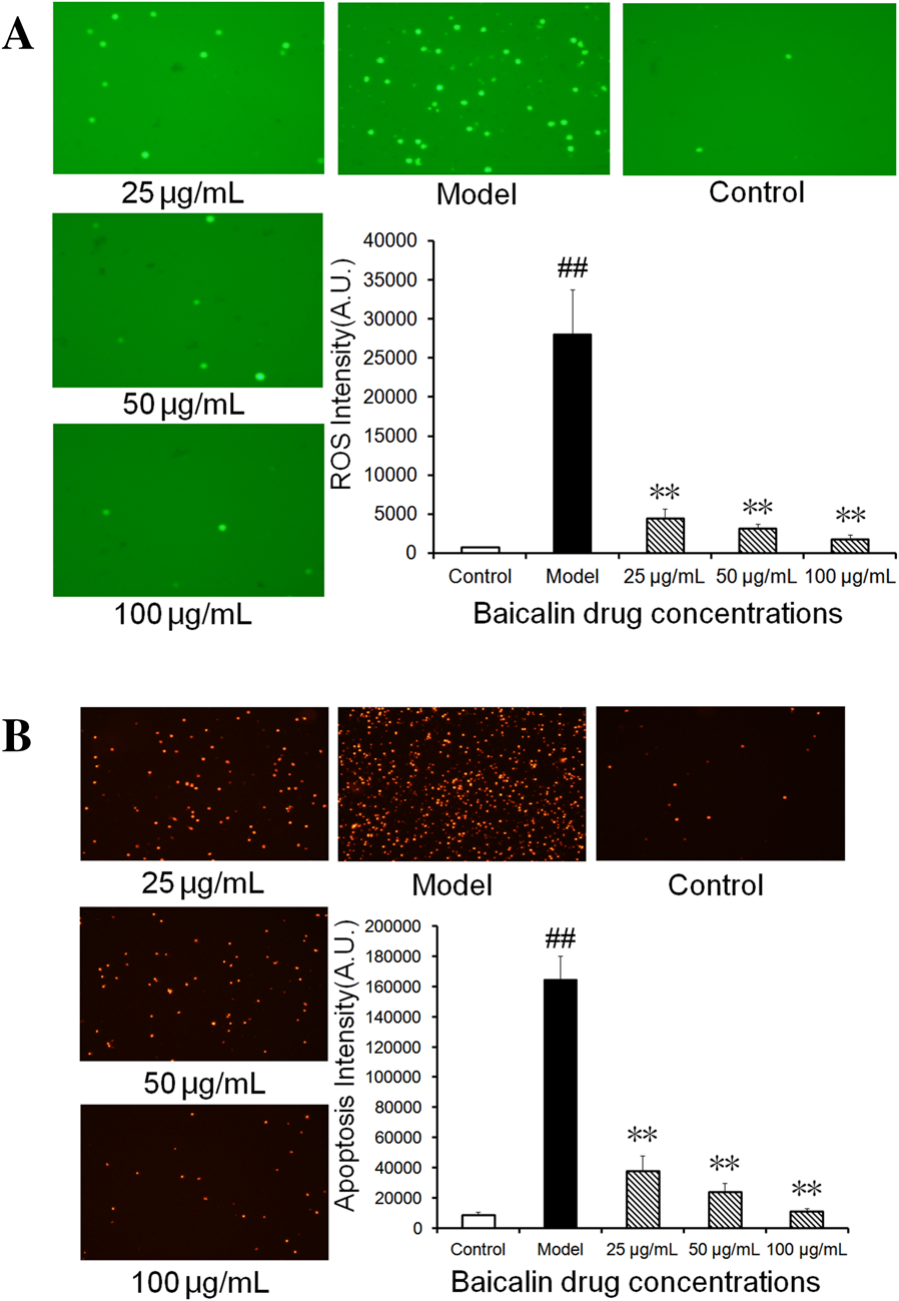
**Effect of baicalin on the production of reactive oxygen species (ROS) and cell apoptosis induced by** ***H. parasuis*** **in peripheral blood monocytes.** **A **1 × 10^6^ of cells were seeded into 24-well plates and treated with various concentrations of baicalin (25, 50, 100 μg/mL) for 2 h. Then 1 × 10^6^ CFU/mL *H. parasuis* were added into the wells and incubated for 3, 6 h respectively. Hence the incubations were washed three times with PBS and stained with 10 μM DCFH-DA and 5 μM DHE for 30 min, respectively. The fluorescence intensities were observed by Fluorescence microscope. **B **1 × 10^6^ of cells were seeded into 24-well plates and treated with various concentrations of baicalin (25, 50, 100 μg/mL) for 2 h. Then 1 × 10^6^ CFU/mL *H. parasuis* were added into the wells and incubated for 3, 6 h respectively. Hence the incubations were washed three times with PBS and stained with Annexin V-FITC and Propidium Iodide, respectively. The fluorescence intensities were observed by Fluorescence microscope. The white bar represents the control group. The black bar represents the model group. The twilled bars represent the baicalin-pretreated groups. ^##^ *p* < 0.01 vs. control. **indicates significance at *p* < 0.01.

Emerging data suggest that ROS plays an important role in regulating inflammasome activation [14]. Therefore, the production of peripheral blood monocyte ROS was determined by measuring mean fluorescence intensity. The peripheral blood monocytes exposed to *H. parasuis* for 3 or 6 h displayed a significantly increase in the ROS generation (*p* < 0.01) (Figure 4A). Meanwhile treatment with baicalin at the concentration of 25 to 100 μg/mL could significantly inhibit the generation of ROS (*p* < 0.01) and the fluorescent microscopy data demonstrated a reduction of intracellular production of ROS in a dose—dependent manner (Figure 4A). On the contrary, whether *H. parasuis* could affect peripheral blood monocyte apoptosis was also investigated. The results show that *H. parasuis* could significantly induce the apoptosis of peripheral blood monocytes following 6 h infection and the production of apoptosis by peripheral blood monocytes induced by *H. parasuis* was inhibited by baicalin at the concentration of 25 to 100 μg/mL (*p* < 0.01) (Figure 4B).

### Effect of baicalin on the activation of NF-κB signaling pathway induced by *H. parasuis* in peripheral blood monocytes

**Figure 5:**
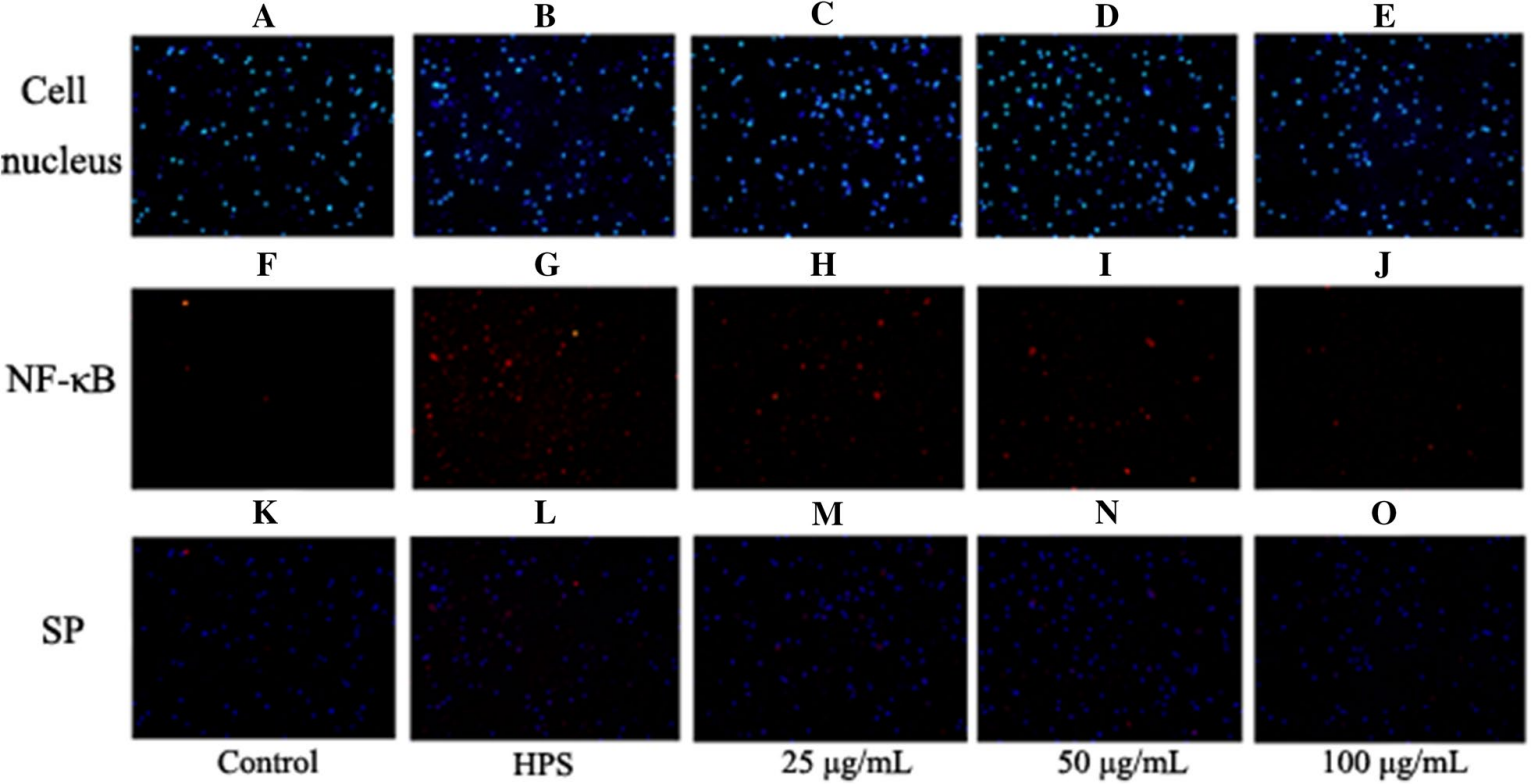
**Effect of baicalin on the activation of NF-κB signaling pathway induced by** ***H. parasuis*** **in peripheral blood monocytes.** **A**–**O** The cells of 1 × 10^7^ seeded on 6-well plates were pretreated with baicalin (25, 50, 100 μg/mL) for 2 h. Then 1.0 × 10^7^ CFU/mL *H. parasuis* was added into the wells. After stimulation of 6 h, the cells were collected and cytoplasmic protein and nucleoprotein were extracted. The expression of NF-κB p65 in cytoplasmic protein and nucleoprotein was measured. SP: superposition.

The levels of the nuclear NF-κB p65 subunit in the monocytes were determined in order to evaluate the effects of baicalin pretreatment on *H. parasuis*-induced monocyte NF-κB signaling. The results demonstrate that the levels of the nuclear NF-κB p65 subunit were significantly increased after *H. parasuis* stimulation at 6 h (*p* < 0.01) (data not shown), suggesting that *H. parasuis* could boost the translocation of the monocyte NF-κB p65 subunit from the cytoplasm to the nucleus. At the same time, immunofluorescence studies also show that nuclear translocation of NF-κB p65 was mainly observed in peripheral blood monocytes (Figure 5L). Meanwhile the effects of baicalin pretreatment on *H. parasuis*-evoked nuclear translocation of NF-κB p65 were analyzed. Interestingly, baicalin pretreatment at 100 μg/mL significantly reduced *H. parasuis*-induced levels of monocyte nuclear NF-κB p65 compared with the pretreatment concentrations of 25 and 50 μg/mL (*p* < 0.01) (Figures 5M–O). Immunofluorescence studies demonstrate that baicalin almost completely inhibited *H. parasuis*-evoked nuclear translocation of NF-κB p65 in peripheral blood monocytes (Figures 5M–O).

### Effect of baicalin on the activation of NLRP3 inflammasome signaling pathway induced by *H. parasuis* in peripheral blood monocytes

**Figure 6:**
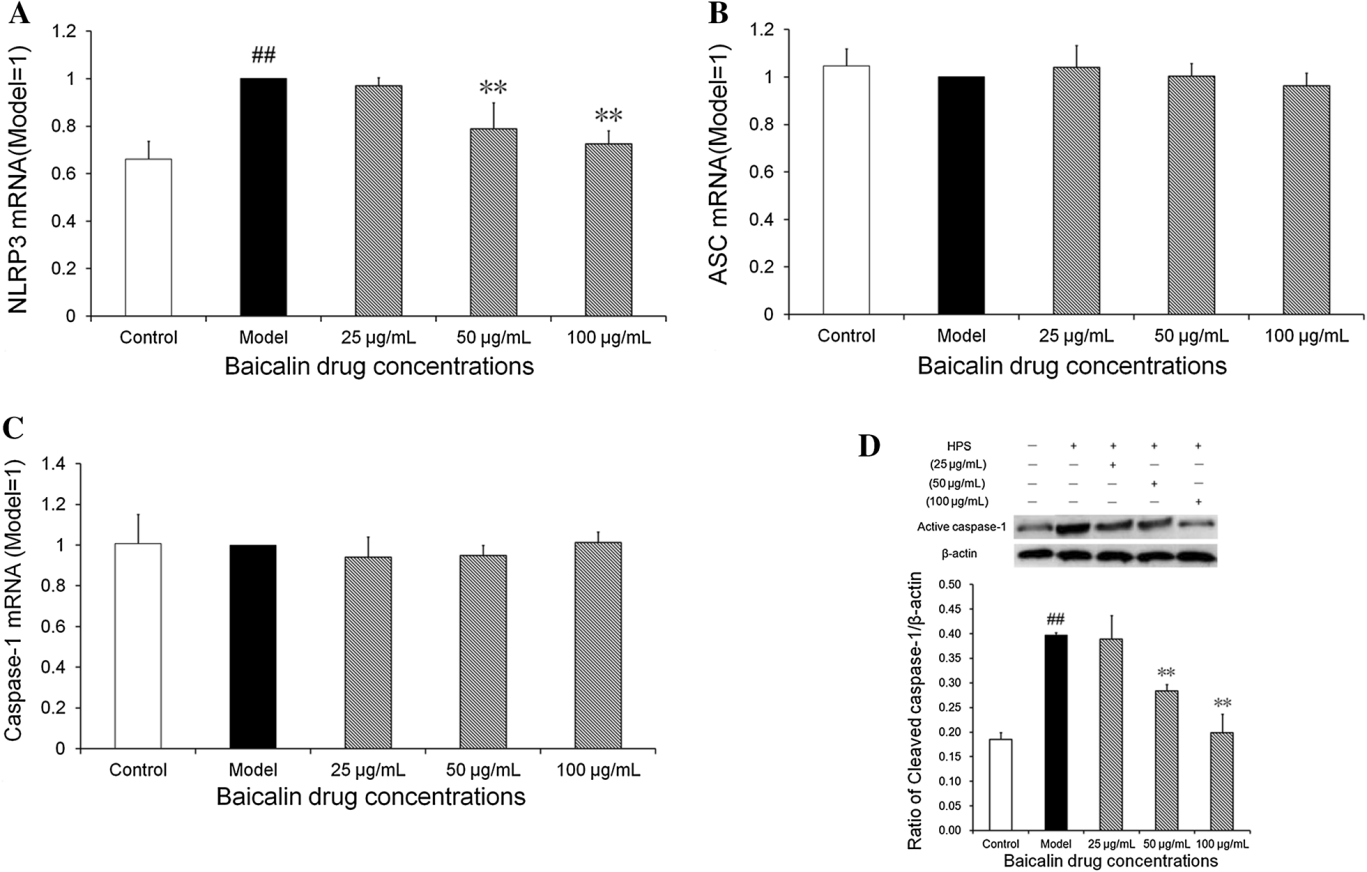
**Effect of baicalin on the activation of NLRP3 inflammasome signaling pathway induced by** ***H. parasuis*** **in peripheral blood monocytes.** **A**–**C** 1 × 10^7^ of cells were pre-treated with baicalin of 25, 50, 100 μg/mL for 2 h. Then 1 × 10^7^ CFU/mL *H. parasuis* were added into the wells and co-incubated for 3, 6 h respectively. Hence the cells were collected and total cellular RNA was extracted and was reverse-transcribed to cDNA. **D** The monocyte cells of 3 × 10^7^ were pretreated with baicalin (25, 50, 100 μg/mL) for 2 h, then 3.0 × 10^7^ CFU/mL *H. parasuis* was added and co-cultured with 5 h, the cells were collected and then the cell protein was extracted for detection of levels of cleaved caspase-1. The white bar represents the control group. The black bar represents the model group. The twilled bars represent the baicalin-pretreated groups. ^##^ *p* < 0.01 vs. control. **indicates significance at *p* < 0.01.

To determine whether NLRP3, ASC and Caspase-1 are activated in peripheral blood monocytes following infection by *H. parasuis*, we evaluated the expression of NLRP3, ASC and caspase-1 at mRNA levels by qRT-PCR. The results indicate that the expression of NLRP3 at the mRNA level in peripheral blood monocytes was significantly up-regulated upon *H. parasuis* infection induction at 6 h compared with the control (*p* < 0.01) (Figure 6A). Treatment with baicalin at the concentration of 50 and 100 μg/mL down-regulated the expression of NLRP3 in contrast to the control (*p* < 0.01) (Figure 6A). Interestingly, infection with *H. parasuis* did not enhance the level of mRNA expression of ASC nor that of caspase-1 (*p* > 0.05) (Figures 6B and C). In addition, the treatment of peripheral blood monocytes with baicalin had no significant effects on the level of mRNA expression of ASC and caspase-1 (*p* > 0.05) (Figures 6B and C).

Furthermore, we also determined the expression of cleaved (active) caspase-1 at the levels of both mRNA and protein by qRT-PCR and Western blotting, respectively, following stimulation with *H. parasuis* for 5 h. The data demonstrate that the level of mRNA of active caspase-1 was significantly increased in the peripheral blood monocytes stimulated by *H. parasuis* compared with the control cells and treatment with baicalin at the concentration of 50 and 100 μg/mL could reduce the expression of active caspase-1, which corresponded to the protein level (*p* < 0.01) (Figure 6D).

## Discussion

Previous studies have suggested that *H. parasuis* could evoke host inflammatory immune responses [37, 38]. However, the mechanism of inflammatory immune responses induced by a signaling pathway is still illusive. Our results show that PMNP infected with *H. parasuis* exhibits transcriptional expression of IL-6, IL-8, IL-10, PGE_2_, COX-2, TNF-α, IL-1β and IL-18. Moreover, baicalin could inhibit the activation of NF-κB and NLRP3 inflammasome signaling pathway triggered by *H. parasuis*, which was first reported in the anti-inflammatory action during *H. parasuis* infection.

In this study, we used piglet blood monocytes isolated from the precaval vein as the cell model. To our best knowledge, this was the first example of a piglet primary immune cell being used for a *H. parasuis* infection study. In general, primary cells are better, more authentic and closely resemble the native environment in studies on the interaction between bacteria and host immune system. Previously established cell lines such as PK cells [10, 39, 40], porcine alveolar macrophages [37, 41], AOC-45 cell lines [42] were used. This newly established bacterial infection model in primary cells provides an excellent system for the study of inflammatory mechanisms during *H. parasuis* and other bacterial infection.

The activation of TLR4 induced by inflammatory stimuli in macrophages such as LPS evokes the release of ROS, which contributes to the NF-κB signiling pathway activation and produces a fast cytokine storm, including spikes in, IL-1β, IL-6 and TNF-α [43, 44]. Inflammatory cytokines such as TNF-α, IL-1β, IL-6 and IL-10 are thought to be important markers that survey inflammatory disease, and they induce tissue injury due to the uncontrolled and prolonged function of these proteins [45, 46]. It has been documented that IL-8, CCL4 and CCL5 can be regulated by NF-κB which is upregulated significantly by *H. parasuis* infection in PK-15 cells, and the activation of the NF-κB pathway is through IκB degradation [9]. In addition, inhibition of NF-κB drastically reduces expression of IL-8 and CCL4 by *H. parasuis* and TLR1, TLR2, TLR4 and TLR6 are required for NF-κB activation in PK-15 cells [10]. Furthermore, porcine Coronin 1A suppresses NF-κB activation by inhibiting the degradation of IκBα and nuclear translocation of p65 in PK-15 cells infected with *H. parasuis* [39]. Thus, we hypothesized that baicalin suppresses inflammation via the inhibition of the NF-κB signaling pathway and we analyzed the expression of p65, TNF-α, IL-6, IL-8, IL-10, COX-2 and PGE_2_ and the production of ROS. Our data demonstrate that the production of TNF-α, IL-6, IL-8, IL-10, COX-2, and PGE_2_ and production of ROS were increased by *H. parasuis* stimulation. Furthermore, ROS production, the translocation of the NF-κB p65 protein into the nucleus, and the levels of TNF-α, IL-6, IL-8, IL-10, COX-2 and PGE_2_ mRNA expression were significantly inhibited in peripheral blood monocytes pretreated with baicalin, suggesting that baicalin could suppress inflammatory responses induced by *H. parasuis*, at least in part, via inhibition of the NF-κB signaling pathway.

Assembly of inflammasome is unique and its production induces many kinds of exogenous and endogenous signals [47]. Inflammasome is also thought to have important effects on the regulation of innate adaptive immune response by taking part in the release of the inflammatory cytokines IL-1β and IL-18 which could launch a large number of biological effects [48]. NLRP3 inflammasome is a key innate immune receptor and mediator in inflammation immune responses. Previous research has reported that NLRP3 plays significant effects on participating in the killing of pathogens and launching wound healing and sterile inflammation [49, 50]. On the contrary, the activation of NLRP3 could also lead to inflammatory tissue injury, including intestinal and systemic inflammatory diseases and hepatic veno-occlusive disease [51, 52]. Macrophages stimulated by ATP induces the levels of ROS, evokes the activation of caspase-1, and releases IL-1β [53]. In addition, some bacteria have been implicated in activation of the NLRP3 inflammasome [54, 55]. For example, *Campylobacter jejuni* could activate NLRP3 inflammasome and induce IL-1β secretion in mouse macrophages without eliciting cell death [56]. Our lab has previously demonstrated that the expression of NLRP3 is stimulated in piglet mononuclear phagocytes induced by LPS and baicalin could inhibit the NLRP3 inflammasome expression [19]. In this study, our data show that the level of IL-1β and IL-18 increased in PMNP after *H. parasuis* infection, and activation of caspase-1 and expression of NLRP3 increases were also determined, inferring that NLRP3 may participate in the initiation of inflammation evoked by *H. parasuis* infection. Although baicalin inhibited the activation of NLRP3 inflammasome, the effects of baicalin on the function of PMNP needs further investigation. So in the future, we plan to conduct further studies on baicalin and the role of NLRP3 in the PMNP of inflammation and pathology changes of *H. parasuis* infection using NLRP3 knockout mice.

Taken together, in our study, we demonstrate for the first time that baicalin has an anti-inflammatory activity, and inhibits the NF-κB signaling pathway and NLRP3 inflammasome activation in PMNP when infected with *H. parasuis*. Our results also indicate that baicalin may possess significant effects on the regulation of innate immune response, which could represent a promising therapeutic strategy for the treatment of *H. parasuis* infection.

## References

[1] Oliveira S, Pijoan C (2004). *Haemophilus parasuis*: new trends on diagnosis, epidemiology and control. Vet Microbiol.

[2] Rapp-Gabrielson VJ, Kocur GJ, Clark JT, Muir SK (1997). *Haemophilus parasuis*: immunity in swine after vaccination. Vet Med.

[3] Kielstein P, Rapp-Gabrielson VJ (1992). Designation of 15 serovars of *Haemophilus parasuis* on the basis of immunodiffusion using heat-stable antigen extracts. J Clin Microbiol.

[4] Aragon V (2013). Exposing serum susceptibility in *Haemophilus parasuis*. Vet J.

[5] Costa-Hurtado M, Aragon V (2013). Advances in the quest for virulence factors of *Haemophilus parasuis*. Vet J.

[6] Costa-Hurtado M, Olvera A, Martinez-Moliner V, Galofré-Milà N, Martínez P, Dominguez J, Aragon V (2013). Changes in macrophage phenotype after infection of pigs with *Haemophilus parasuis* strains with different levels of virulence. Infect Immun.

[7] Bouchet B, Vanier G, Jacques M, Gottschalk M (2008). Interactions of *Haemophilus parasuis* and its LOS with porcine brain microvascular endothelial cells. Vet Res.

[8] Bouchet B, Vanier G, Jacques M, Auger E, Gottschalk M (2009). Studies on the interactions of *Haemophilus parasuis* with porcine epithelial tracheal cells: limited role of LOS in apoptosis and pro-inflammatory cytokine release. Microb Pathog.

[9] Chen Y, Jin H, Chen P, Li Z, Meng X, Liu M, Li S, Shi D, Xiao Y, Wang X, Zhou Z, Bi D, Zhou R (2012). *Haemophilus parasuis* infection activates the NF-κB pathway in PK-15 cells through IκB degradation. Vet Microbiol.

[10] Chen Y, Liu T, Langford P, Hua K, Zhou S, Zhai Y, Xiao H, Luo R, Bi D, Jin H, Zhou R (2015). *Haemophilus parasuis* induces activation of NF-κB and MAP kinase signaling pathways mediated by toll-like receptors. Mol Immunol.

[11] Vallabhapurapu S, Karin M (2009). Regulation and function of NF-κB transcription factors in the immune system. Annu Rev Immunol.

[12] Mussá T, Rodríguez-Cariño C, Sánchez-Chardi A, Baratelli M, Costa-Hurtado M, Fraile L, Domínguez J, Aragon V, Montoya M (2012). Differential interactions of virulent and non-virulent *H. parasuis* strains with naïve or swine influenza virus pre-infected dendritic cells. Vet Res.

[13] Lukens JR, Gross JM, Kanneganti TD (2012). IL-1 family cytokines trigger sterile inflammatory disease. Front Immunol.

[14] Latz E, Xiao TS, Stutz A (2013). Activation and regulation of the inflammasomes. Nat Rev Immunol.

[15] Liu P, Xie Q, Wei T, Chen Y, Chen H, Shen W (2015). Activation of the NLRP3 inflammasome induces vascular dysfunction in obese OLETF rats. Biochem Biophys Res Commun.

[16] Shao BZ, Xu ZQ, Han BZ, Su DF, Liu C (2015). NLRP3 inflammasome and its inhibitors: a review. Front Pharmacol.

[17] Phongsisay V (2016). The immunobiology of *Campylobacter jejuni*: innate immunity and autoimmune diseases. Immunobiology.

[18] Liu Y, Yao W, Xu J, Qiu Y, Cao F, Li S, Yang S, Yang H, Wu Z, Hou Y (2015). The anti-inflammatory effects of acetaminophen and N-acetylcysteine through suppression of the NLRP3 inflammasome pathway in LPS-challenged piglet mononuclear phagocytes. Innate Immun.

[19] Ye C, Li S, Yao W, Xu L, Qiu Y, Liu Y, Wu Z, Hou Y (2016). The anti-inflammatory effects of baicalin through suppression of NLRP3 inflammasome pathway in LPS-challenged piglet mononuclear phagocytes. Innate Immun.

[20] Granata S, Masola V, Zoratti E, Scupoli MT, Baruzzi A, Messa M, Sallustio F, Gesualdo L, Lupo A, Zaza G (2015). NLRP3 inflammasome activation in dialyzed chronic kidney disease patients. PLoS One.

[21] Zhong Z, Zhai Y, Liang S, Mori Y, Han R, Sutterwala FS, Qiao L (2013). TRPM2 links oxidative stress to NLRP3 inflammasome activation. Nat Commun.

[22] Guarda G, Zenger M, Yazdi AS, Schroder K, Ferrero I, Menu P, Tardivel A, Mattmann C, Tschopp J (2011). Differential expression of NLRP3 among hematopoietic cells. J Immunol.

[23] Wen H, Ting JP, O’Neill LA (2012). A role for the NLRP3 inflammasome in metabolic diseases–did Warburg miss inflammation?. Nat Immunol.

[24] Mulay SR, Kulkarni OP, Rupanagudi KV, Migliorini A, Darisipudi MN, Vilaysane A, Muruve D, Shi Y, Munro F, Liapis H, Anders HJ (2013). Calcium oxalate crystals induce renal inflammation by NLRP3-mediated IL-1β secretion. J Clin Invest.

[25] Jeong HS, Gu GE, Jo AR, Bang JS, Yun HY, Baek KJ, Kwon NS, Park KC, Kim DS (2015). Baicalin-induced Akt activation decreases melanogenesis through downregulation of microphthalmia-associated transcription factor and tyrosinase. Eur J Pharmacol.

[26] Shieh DE, Liu LT, Lin CC (2000). Antioxidant and free radical scavenging effects of baicalein, baicalin and wogonin. Anticancer Res.

[27] Chen H, Xu Y, Wang J, Zhao W, Ruan H (2015). Baicalin ameliorates isoproterenol-induced acute myocardial infarction through iNOS, inflammation and oxidative stress in rat. Int J Clin Exp Pathol.

[28] Chu M, Xu L, Zhang MB, Chu ZY, Wang YD (2015). Role of baicalin in anti-influenza virus A as a potent inducer of IFN-Gamma. Biomed Res Int.

[29] Moghaddam E, Teoh BT, Sam SS, Lani R, Hassandarvish P, Chik Z, Yueh A, Abubakar S, Zandi K (2014). Baicalin, a metabolite of baicalein with antiviral activity against dengue virus. Sci Rep.

[30] Wang T, Shi G, Shao J, Wu D, Yan Y, Zhang M, Cui Y, Wang C (2015). In vitro antifungal activity of baicalin against *Candida albicans* biofilms via apoptotic induction. Microb Pathog.

[31] Yu Y, Pei M, Li L (2015). Baicalin induces apoptosis in hepatic cancer cells in vitro and suppresses tumor growth in vivo. Int J Clin Exp Med.

[32] Dong SJ, Zhong YQ, Lu WT, Li GH, Jiang HL, Mao B (2015). Baicalin inhibits lipopolysaccharide-induced inflammation through signaling NF-κB pathway in HBE16 airway epithelial cells. Inflammation.

[33] Liu T, Dai W, Li C, Liu F, Chen Y, Weng D, Chen J (2015). Baicalin alleviates silica-induced lung inflammation and fibrosis by inhibiting the Th17 response in C57BL/6 mice. J Nat Prod.

[34] Min W, Ahmad I, Chang ME, Burns EM, Qian Q, Yusuf N (2015). Baicalin protects keratinocytes from toll-like receptor-4 mediated DNA damage and inflammation following ultraviolet irradiation. Photochem Photobiol.

[35] Chen G, Feng W, Zhang S, Bian K, Yang Y, Fang C, Chen M, Yang J, Zou X (2015). Metformin inhibits gastric cancer via the inhibition of HIF1α/PKM2 signaling. Am J Cancer Res.

[36] Shafagh M, Rahmani F, Delirezh N (2015). CuO nanoparticles induce cytotoxicity and apoptosis in human K562 cancer cell line via mitochondrial pathway, through reactive oxygen species and P53. Iran J Basic Med Sci.

[37] Kavanová L, Prodělalová J, Nedbalcová K, Matiašovic J, Volf J, Faldyna M, Salát J (2015). Immune response of porcine alveolar macrophages to a concurrent infection with porcine reproductive and respiratory syndrome virus and *Haemophilus parasuis* in vitro. Vet Microbiol.

[38] Macedo N, Rovira A, Torremorell M (2015). *Haemophilus parasuis*: infection, immunity and enrofloxacin. Vet Res.

[39] Liu C, Wang Y, Zhang H, Cheng S, Charreyre C, Audonnet JC, Chen P, He Q (2014). Porcine coronin 1A contributes to nuclear factor-kappa B (NF-κB) inactivation during *Haemophilus parasuis* infection. PLoS One.

[40] Chen Y, Zhou S, Hua K, Xiao H, Li Z, Liu M, Luo R, Bi D, Zhou R, Jin H (2015). *Haemophilus parasuis* infection activates chemokine RANTES in PK-15 cells. Mol Immunol.

[41] Zhou S, He X, Xu C, Zhang B, Feng S, Zou Y, Li J, Liao M (2014). The outer membrane protein P2 (OmpP2) of *Haemophilus parasuis* induces proinflammatory cytokine mRNA expression in porcine alveolar macrophages. Vet J.

[42] Frandoloso R, Pivato M, Martínez-Martínez S, Rodríguez-Ferri EF, Kreutz LC, Martín CB (2013). Differences in *Haemophilus parasuis* adherence to and invasion of AOC-45 porcine aorta endothelial cells. BMC Vet Res.

[43] Verstrepen L, Bekaert T, Chau TL, Tavernier J, Chariot A, Beyaert R (2008). TLR-4, IL-1R and TNF-R signaling to NF-kappa B: variations on a common theme. Cell Mol Life Sci.

[44] Ka SM, Kuoping Chao L, Lin JC, Chen ST, Li WT, Lin CN, Cheng JC, Jheng HL, Chen A, Hua KF (2016). A low toxicity synthetic cinnamaldehyde derivative ameliorates renal inflammation in mice by inhibiting NLRP3 inflammasome and its related signaling pathways. Free Radic Biol Med.

[45] Li W, Suwanwela NC, Patumraj S (2016). Curcumin by down regulating NF-kB and elevating Nrf2, reduces brain edema and neurological dysfunction after cerebral I/R. Microvasc Res.

[46] Kaphalia L, Kalita M, Kaphalia BS, Calhoun WJ (2016). Effects of acute ethanol exposure on cytokine production by primary airway smooth muscle cells. Toxicol Appl Pharmacol.

[47] Broz P (2015). Inflammasome assembly: the wheels are turning. Cell Res.

[48] Benetti E, Chiazza F, Patel NS, Collino M (2013). The NLRP3 inflammasome as a novel player of the intercellular crosstalk in metabolic disorders. Mediators Inflamm.

[49] Xiang Y, Wang X, Yan C, Gao Q, Li SA, Liu J, Zhou K, Guo X, Lee W, Zhang Y (2013). Adenosine-5′-triphosphate (ATP) protects mice against bacterial infection by activation of the NLRP3 inflammasome. PLoS One.

[50] Weinheimer-Haus EM, Mirza RE, Koh TJ (2015). Nod-like receptor protein-3 inflammasome plays an important role during early stages of wound healing. PLoS One.

[51] Qiao J, Huang Y, Xia Y, Chu P, Yao H, Xu L, Qi K, Liu Y, Xu K, Zeng L (2015). Busulfan and cyclosphamide induce liver inflammation through NLRP3 activation in mice after hematopoietic stem cell transplantation. Sci Rep.

[52] Filardy AA, He J, Bennink J, Yewdell J, Kelsall BL (2016). Posttranscriptional control of NLRP3 inflammasome activation in colonic macrophages. Mucosal Immunol.

[53] Cruz CM, Rinna A, Forman HJ, Ventura AL, Persechini PM, Ojcius DM (2007). ATP activates a reactive oxygen species-dependent oxidative stress response and secretion of proinflammatory cytokines in macrophages. J Biol Chem.

[54] Toma C, Higa N, Koizumi Y, Nakasone N, Ogura Y, McCoy AJ, Franchi L, Uematsu S, Sagara J, Taniguchi S, Tsutsui H, Akira S, Tschopp J, Núñez G, Suzuki T (2010). Pathogenic Vibrio activate NLRP3 inflammasome via cytotoxins and TLR/nucleotide-binding oligomerization domain-mediated NF-kappa B signaling. J Immunol.

[55] Zhang X, Cheng Y, Xiong Y, Ye C, Zheng H, Sun H, Zhao H, Ren Z, Xu J (2012). Enterohemorrhagic Escherichia coli specific enterohemolysin induced IL-1β in human macrophages and EHEC-induced IL-1β required activation of NLRP3 inflammasome. PLoS One.

[56] Bouwman LI, de Zoete MR, Bleumink-Pluym NM, Flavell RA, van Putten JP (2014). Inflammasome activation by *Campylobacter jejuni*. J Immunol.

